# On the Art of Binocular Rivalry

**DOI:** 10.1177/20416695211053877

**Published:** 2021-11-25

**Authors:** Nicholas J. Wade

**Affiliations:** Psychology, University of Dundee, Dundee DD1 4HN, UK

**Keywords:** anaglyphs, binocular art, contour rivalry, lustre, stereoscopic vision, portraits, photography, graphics

## Abstract

Binocular rivalry has a longer descriptive history than stereoscopic depth perception both of which were transformed by Wheatstone's invention of the stereoscope. Thereafter, artistic interest in binocular vision has been largely confined to stereopsis. A brief survey of research on binocular contour rivalry is followed by anaglyphic examples of its expression as art. Rivalling patterns can be photographs, graphics, and combinations of them. In addition, illustrations of binocular lustre and interactions between rivalry and stereopsis are presented, as are rivalling portraits of some pioneers of the science and art of binocular vision. The question of why a dynamic process like binocular rivalry has been neglected in visual art is addressed.

## Introduction

The investigation of vision with two eyes is one of the oldest areas of vision research with attention concentrated on single and double vision (see Howard and Rogers, 1995; Wade, 1998). Stereoscopic depth perception entered the experimental scene with the announcement of the stereoscope (Wheatstone, 1838) and it has been studied intensively thereafter. The history of research on binocular vision has been dominated by two concepts that were advanced at about the same time. Porta (1593) proposed that the world is seen singly because only one eye is used at one time: signals from the other eye are suppressed. Aguilonius (1613) presented an alternative interpretation: similar signals from the two eyes are fused to yield single vision. This is essentially the contrast between binocular competition and cooperation. Studies of one have tended to be conducted independently of the other (see Wolfe, 1986).

Long before the invention of the stereoscope, rivalry between dissimilar images in each eye was described and devices were made for determining some of its characteristics (see Wade and Ngo, 2013). The principal topic was how different colours presented to the eyes were perceived but some studies did examine rivalry between contours. Both colour and contour rivalry could be investigated more conveniently with the aid of a stereoscope, as was described by Wheatstone (1838). Photography was announced to the public in the following year and Wheatstone (1852) recognized the assistance that it would provide for stereoscopy. Since then most stereoscopic art has been based on photography, but little of this has intentionally involved binocular rivalry.

Among the different types of stereoscope were those that presented different coloured stimuli viewed through similarly coloured filters; they became called anaglyphs (see Wade, 2021a). The coloured glasses act as filters so that contours of the same colour (say red) are not seen whereas those of the other (cyan) appear black; this applies irrespective of the colour vision of the observer. The anaglyphs in this article are designed to be viewed with red/cyan filters. Anaglyphs of scenes producing stereoscopic depth require an appropriate pairing of the coloured components and the red/cyan filters; reversing the filters does not reverse the depth. Anaglyphs of rivalling patterns can be viewed with either arrangement of colours and filters. Readers are encouraged to view the anaglyphs with both arrangements of the colour filters, that is, red/left eye and cyan/right eye as well as cyan/left eye and red/right eye. The main difference will be a consequence of any eye dominance that might exist in the observer.

Stereoscopic art was mainly confined to photography although abstract works have increased since the introduction of random dot stereograms (see Wade, 2021b). Examples of art based on binocular rivalry have been sparse in comparison. The Romanian visual scientist Liviu Iliescu has produced many examples of what he calls bioptical art; they are paired drawings and paintings which can be viewed by over-convergence thereby generating binocular rivalry (see Iliescu, 2015). Some photographers are also using binocular rivalry in their compositions. For example, Antonio McAfee enlists anaglyphic images in his examinations of historical photographs of middle-class African Americans. He reprocesses old portraits often combining them with their left-right reversals to produce symmetrical anaglyphs which are viewed with red/cyan glasses. The anaglyphic portraits can be partially masked with yet other images superimposed on them (see http://printcenter.org/93rd/mcafee/). One of the few artists explicitly espousing rivalry art based on binocular lustre is Yuki Maruyama. She makes large installations painted in red and cyan and provides red/cyan glasses to view them (see http://www.adobebooks.com/blog/2020/1/1/yuki-maruyama). The juxtaposition of the large areas of the two colours induces a variety of visual effects and these are enhanced with the lustrous reversals seen when wearing the red/cyan glasses.

Artistic combinations of stereoscopic depth and rivalry are even rarer. Colvin (2009) paints over three-dimensional structures and photographs the final scene from the position in which all the painted contours are in appropriate alignment. When viewing the photographs, the solid scene is initially overlooked and pictorial flatness dominates perception. By adopting two viewpoints, neither of which will yield perfect alignment between the contours painted on the solid objects, retinal disparity is introduced. Depth derived from disparity vies with pictorial depth, so that the works are not narrowly stereoscopic but they display competition between the pictorial and binocular cues to depth. Rivalry is introduced between selected elements, usually in the peripheral parts of the scene within each stereoscopic image. For example, a candle flame can be visible to one eye but the candle remains unlit in the other. However, central rivalry is also explored by Colvin who has made artworks that return to the origins of stereoscopy. In one, there is rivalry between an indistinct portrait of Brewster in one eye and a shadowy Wheatstone in the other, each accompanying a celebrated drawing by Chimenti (see Wade, 2003). When viewed in a stereoscope, Wheatstone and Brewster can be seen hovering in symbolic rivalry relative to the Chimenti drawing (see Wade, 2009, Figure 4).

## Binocular Rivalry

**Figure 1.**
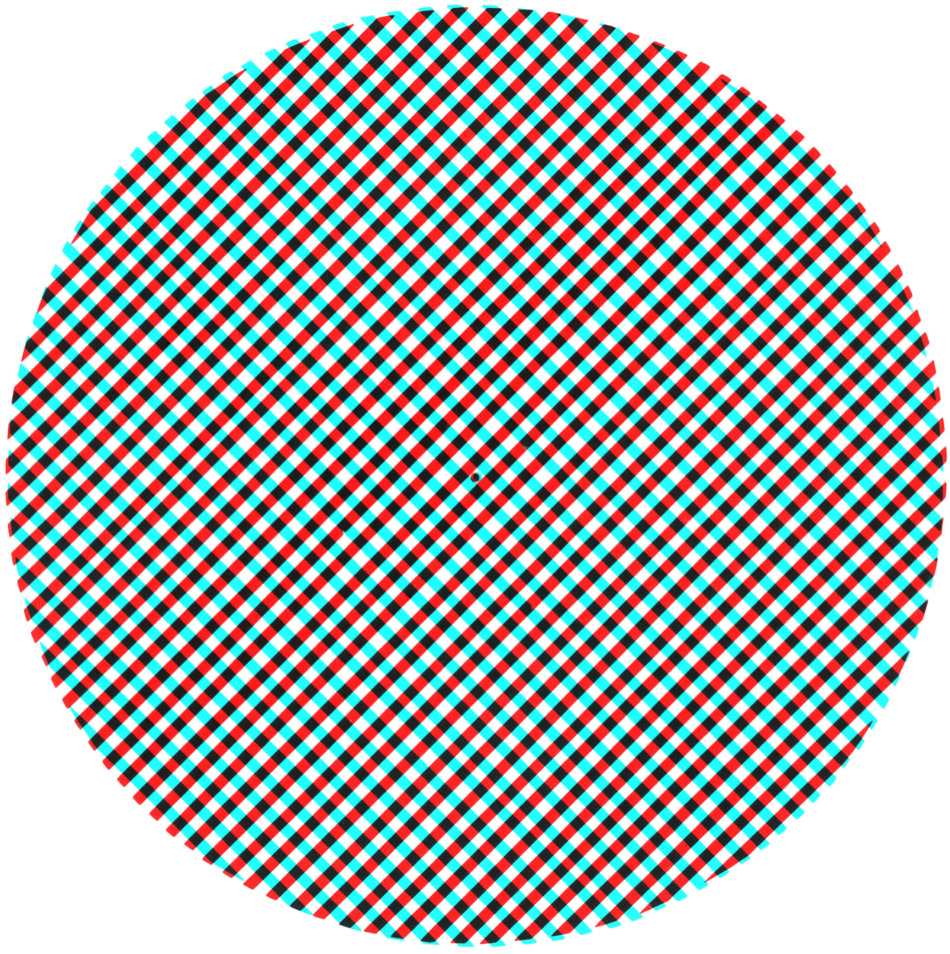
A rivalry pattern consisting of orthogonal gratings. Fixating on the central dot while looking through red/cyan filters will result in binocular rivalry. Reversing the filters will indicate whether one eye has a marked dominance.

Binocular contour rivalry is more evident and compelling than colour rivalry and it can readily be observed with different stimuli in a stereoscope. Wheatstone (1838) examined rivalry between the letters A and S each surrounded by similar circles (see Wade, 2019). Letters were recognised as complex patterns and simpler stimuli were soon enlisted. One of the first systematic studies of rivalry was published by Panum (1858). The orthogonal grating stimuli he introduced have dominated the study of rivalry ever since. Panum drew attention to the dynamic variations and to the mixtures or composites that are seen and he sought to interpret the phenomenon in physiological terms rather than the psychological factors proposed by Wheatstone (1838) and later by Helmholtz (1867). Rivalry patterns now used are like that shown in Figure 1. Pattern fluctuations can be seen without the filters or with one eye and they are referred to as monocular rivalry (see O'Shea et al., 2017). Binocular rivalry involves periods of local or global dominance of one or other orientation whereas monocular rivalry is generally experienced as fluctuations in the distinctiveness of the gratings (Wade, 1975).

Wundt (1862) was quick to appreciate the import of Panum's work and incorporated it into his book on sensory perception. In his survey of binocular vision Wundt included a section on rivalry of perception and presented figures similar to those employed by Panum, like pairs of vertical and horizontal lines as well as vertical and horizontal gratings. Wundt drew attention to the effects of eye movements on the visibility of such patterns. He also emphasised the mixtures that are visible under normal conditions of viewing. He interpreted rivalry in terms of alternations of attention. It is significant that Wundt modified Panum's ‘rivalry between the visual fields’ to ‘rivalry of perception’ because it reflected a distinction that remains to this day: is rivalry a psychological or a physiological process? Panum considered that it was physiological whereas Wheatstone, Helmholtz and Wundt maintained that it was psychological. Helmholtz (1867) discussed rivalry in some detail and also emphasised that changing, complex mixtures of the two stimuli tend to be visible most of the time with only occasional periods in which the stimulus in one eye alone dominates. Like Wundt, Helmholtz placed great importance on eye movements in rivalry and made a modification to Panum's orthogonal gratings configurations: he placed two small squares at the centre of both gratings to facilitate common fixation by each eye (see Wade, 2021c). The crossed diagonal figure was used by Helmholtz to support his theory that rivalry is a psychological rather than a physiological process because he could control which stimulus was visible.

Rivalry between photographs of faces was examined by Galton (1878, 1879). The impetus for his experiments on composite faces stemmed from presenting different photographic portraits in a stereoscope: “At first, for obtaining pictorial averages I combined pairs of portraits with a stereoscope, with more or less success” (Galton, 1908, p. 259). When Galton devised a method for making multiple exposures of different faces on a single photographic plate, he dispensed with the use of the stereoscope. At around the time of Galton's initial experiments, similar stereoscopic combinations of different portraits were conducted independently and they were communicated to Galton's half-cousin, Charles Darwin, who passed them on to Galton (see Wade, 2016b). The purpose of combining different portraits was not to investigate binocular rivalry but to derive some composite average of human types. Nonetheless, it is clear from Galton's description that rivalry did occur and it was for this reason that composite photographs were preferred. Paired photographic stimuli did not have the impact on studies of binocular rivalry that they did for stereoscopic vision.

From the end of the 19th century, neural theories of consciousness based on rivalry experiments were advanced by Breese (1899) who introduced measurements of the periods of visibility and the frequencies of dominance of rivalling stimuli. These were two squares with 45° and 135° black lines on them; they were on backgrounds that were either green or red, or they were both the same colour. He found no differences in either the predominance durations or the periods of visibility under these conditions. It should, however, be noted that the comparisons were taken from different experiments and were based upon his own observations alone. There is a further problem in interpreting Breese's findings: he used gratings of unspecified visual subtense, and recorded the phenomenal alternation between the two gratings. In view of the history of experiments on contour rivalry it is unlikely that there was simply alternation between the two gratings. Almost all previous investigators noted that fragments of the two monocular stimuli appear simultaneously in different parts of the field. It has since been found that these fragments or composites are visible for around 30% of the observation period for rivalry between gratings (Wade, 1974). Perhaps Breese observed such composites and categorised them in terms of the dominance of one of the monocular fields, although he made no mention of this.

Along with examining the effects of motor inhibition on memory, Breese used binocular rivalry as a paradigm to examine the inhibition of sensations and argued that consciousness had a sensorimotor basis. More importantly, he made the first quantitative measures of binocular rivalry. He examined: (i) the effect of stimulus strength changes (e.g., motion, size, luminance) on perceptual predominance and alternation rate; (ii) individual variation in alternation rate; (iii) the effect of unilateral motor activity on predominance; (iv) rivalry between after-images and their slower alternation rate compared to real stimuli; and (iv) the phenomenon of monocular rivalry. He also investigated the influence of willpower on binocular rivalry. Subjects could voluntarily hold attention on one image with the (inadvertent) use of eye movements, but without eye movements such voluntary control was limited. Breese argued that binocular rivalry could not be explained by purely mental conditions because complete control over the alternations could not be demonstrated.

Correspondingly, physical conditions such as retinal adaptation could not solely explain the effect of different brightness levels on rivalry rate, which may have also been due to greater attention directed towards the brighter image. Instead, he concluded that the phenomenon “would be at once ‘psychical’ and ‘physiological’ in that it is dependent upon central processes, and is affected by the nature of motor adaptations” (Breese, 1899, p. 48). Breese (1917) subsequently elaborated on the distinction between consciousness and attention during rivalry, along with postulating their associated activity in the brain.

Ten years after his original study, Breese (1909) repeated the rivalry experiments on himself and noted that his alternation rate was almost identical. This within-individual retest reliability of binocular rivalry rate was also reported by others, as was his earlier finding of individual variation in alternation rate. The quantitative rivalry experiments conducted by Breese and his interpretation of their findings reflected the broader development of psychology into a scientific discipline as well as interest in the neural basis of attention and consciousness.

**Figure 2.**
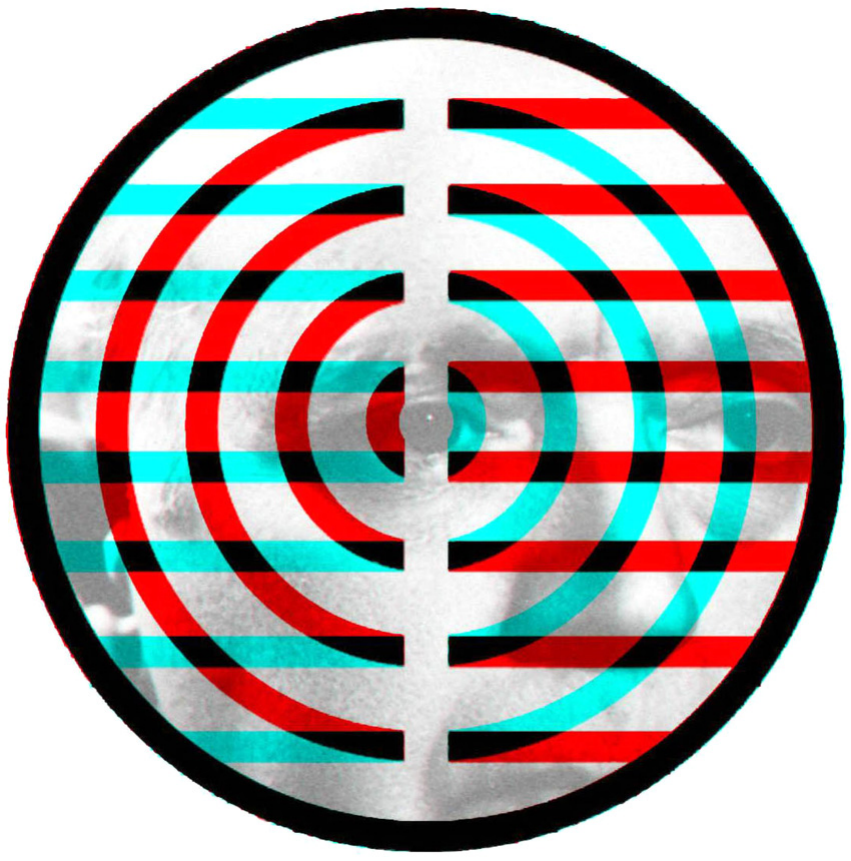
*Emilio Diaz-Caneja and his rivalry pattern* by Nicholas Wade.

Experimental studies of binocular rivalry were relatively sparse in the first half of the 20th century but there were some significant studies. One by Diaz-Caneja (1928) concerned the influence of organization in rivalry; his article was largely forgotten until it was translated over 70 years later (Alais et al., 2000). Diaz-Caneja used rivalry patterns in which each half was formed from red horizontal lines and green semicircles (Figure 2). He found that rivalry was not solely between the eyes but also between the patterns. That is, horizontal lines or concentric circles were also seen so that the components from different eyes were integrated: “At a particular moment, the lines and circles are mixed; an instant after, we can see lines everywhere or circles everywhere; more rarely, we see either the right half or the left half of the card” (Alais et al., 2000, p. 1444). Diaz-Caneja was demonstrating that rivalry can be between figures, parts of which are in opposite eyes as well as by figures in each eye. This is an issue that has continued to reverberate throughout rivalry research (Blake & Logothetis, 2002).

The pace of research on rivalry quickened in the second half of the century. It was boosted in the 1960s by three researchers – Robert Fox in America, William (Pim) Levelt in The Netherlands, and Paul Whittle in Britain (see Blake, 2005). All brought an added precision to the recording and analysis of rivalry sequences. Levelt (1965) presented propositions regarding rivalry which have stood the test of time (see Brascamp et al., 2015). A key concept was that the strength of the stimuli in each eye determined the characteristics of the rivalry that ensued. This could be supported by examining the individual rivalry dominance and suppression durations and describing the distributions mathematically.

In common with Levelt, Fox (2005; Blake and Fox, 1974) made detailed analyses of the durations of dominance and suppression in sequences of rivalry. He also drew attention to the piecemeal aspects of binocular rivaly, particularly when large stimuli are presented to an observer, as well as the influence of stimulus motion. He developed techniques that presented brief probes to an eye when the stimulus to that eye was suppressed and found that visual sensitivity was reduced, unlike the same presentation when the pattern was dominant. Moreover, quite marked changes in the stimulus presented to the suppressed eye can go undetected suggesting that rivalry is between the eyes rather than the particular stimuli. Fox introduced a technique that has had considerable experimental traction: can a suppressed stimulus influence the characteristics of a pattern presented subsequently? More specifically, can a visual aftereffect be induced by a pattern in the suppressed eye? In general, the answer from many studies has been in the positive (O'Shea & Crassini, 1981; Wade, 1980; Wade & Wenderoth, 1978).

A modification of the view that rivalry is a low level aspect of binocular interaction was provided by Whittle (1965; Whittle et al., 1968); he examined aspects of figural organization and contrast in binocular rivalry. That is, he sought to distinguish between local and global interactions in rivalry. For example, when segments of a figure viewed by different eyes engage in rivalry they do so in a similar way to the segments presented to the same eye. This applies whether the stimuli are viewed in a stereoscope or as afterimages (Wade, 1973). The issue of local and global interactions features in a wide range of visual phenomena and their extension to binocular vision was a significant advance. A novel approach to this question was examined by Kovács et al. (1996); they compared rivalry between well-defined patterns (text on a jungle scene in one eye and a monkey's face in the other) with patchwork pairing of these elements. In the latter there were periods in which the text and face were separately visible indicating that pattern coherence occurs between the eyes and can engage in rivalry.

Much more has been learned about responses to patterns in the mammalian visual cortex since the 1960s as well as novel techniques for recording from the human brain. Accordingly, concerted efforts have been made to link binocular rivalry using a wider range of stimulus patterns with measures of cortical activity. Recordings from single cells in monkey visual cortex can be related to stimuli they see (Leopold & Logothetis, 1996; Logothetis et al., 1996). A combination introduced by Tong and colleagues has been used extensively to illustrate such links (Tong, 2005; Tong et al., 1998); it consists of a picture of a face presented to one eye and of a house to the other. They found that the periods when the face was dominant corresponded to increased activity in a brain region known to be related to face processing (fusiform face area) whereas those when the house was dominant led to similar effects in an area concerned with coding location (parahippocampal place area).

Much of the subsequent research has examined the distinction between theories proposing a low level basis for binocular rivalry or whether higher level processes are involved in it (Alais & Blake, 2005; Blake, 2017). Investigations of binocular rivalry have broadened enormously in the last decades and much attention is directed at determining the neurophysiological underpinnings of dominance and suppression as well as their interplay. Increasingly, computational models of the perceptual oscillations have been developed. Moreover, advances in computer controlled stimulus presentation and analysis have extended the subtleties of the experimental manipulations that are available. Individual differences in the temporal dynamics of rivalry has also come to the forefront, either by relating the pattern of rivalry sequences to known dimensions of personality (like introversion/extraversion) or to underlying physical conditions (like vestibular disorders) (Ngo et al., 2013). Rivalry has been used as a way of investigating consciousness and its neural correlates (Blake et al., 2014; Miller, 2013). Indeed it has been said that: “Binocular rivalry is a popular tool in the scientific study of consciousness because it dissociates stable, unchanged, visual stimulation from fluctuations in visual awareness” (Klink et al., 2013, p. 323). Reviews of this research can be found in numerous articles and books (Alais & Blake, 2005, 2015; Miller, 2013) and O'Shea has assembled a bibliography of rivalry research up to 2001 (https://sites.google.com/site/oshearobertp/publications/binocular-rivalry-bibliography).

## Rivalry Art

Following the invention of stereoscopes there has been a growing interest in producing binocular art, either from paired photographs or less commonly from paired drawings or paintings (see Wade, 2021b). Surprisingly, much less attention has been directed to art involving binocular rivalry considering that the visual dynamics of rivalry are more striking than the subtleties of stereoscopic depth perception. The visual transformations that occur during binocular rivalry are easy to experience but difficult to describe. This applies particularly to the changes that take place when complex patterns compete with one another. Scientists have tried to overcome this by using very simple patterns like gratings but few artists have revelled in the dynamic variety that is a consequence of processes occurring in the brain rather than on the pictorial surface. This was so even in Op Art where emphasis was placed on visual variation with static patterns although some examples of rivalling patterns were presented in *The art and science of visual illusions* (Wade, 1982). At that time publishers were reluctant to print coloured figures and although anaglyphs were initially proposed for inclusion in the book they were not so published. In its place a mirror method was recommended for viewing the paired patterns, essentially like one proposed by Brewster (1851). Paired geometrical designs are viewed with a single mirror placed between the eyes:

The mirror should be placed with its upper side aligned with the nose and the centre of the forehead and its base directed between the two patterns. If the reflecting surface is towards the right side then the reflected image will undergo a left-right reversal with respect to the printed pattern, and it will also appear slightly smaller than the left pattern (provided the printed dimensions of the two are the same). The direction from which the reflected image appears to come can be changed by moving the bottom of the image to the left or right. It is possible to view the left pattern directly with the left eye and the right, reflected, image can be adjusted to be in the same visual direction. That is, the two patterns appear to occupy the same positions in space. (Wade, 1982, p. 151)

**Figure 3.**
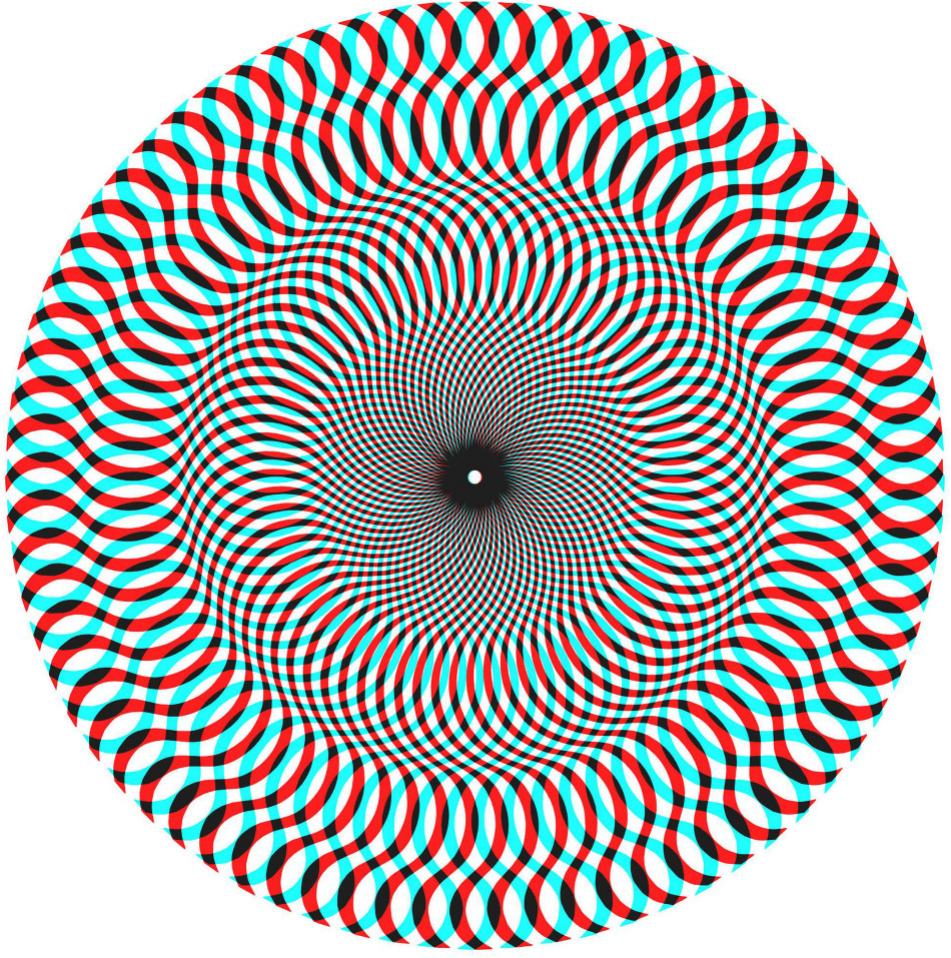
*Rivalling radiations* by Nicholas Wade (an anaglyph based on Figure 1.8.10 from Wade, 1982). The component monocular designs are themselves visually unstable and are also seen as implying impossible depths.

Anaglyph techniques and printing technologies have advanced enormously since that time and an anaglyph of one of the rivalry figures from the book is shown in Figure 3.

One factor that has inhibited artists from employing rivalry in their work is the need for some binocular viewing device. However, this same argument applies to stereoscopic art which has a vibrant history spanning back to the invention of the stereoscope. The options for rivalry art are broader than those for stereo art and they can be expressed through photography as well as graphics. Indeed, binocular contour rivalry with anaglyphs presents an additional advantage because the component patterns can interact with one another in monocular vision so that many visual possibilities arise; they can also be projected onto a screen so that the rivalry can be experienced by many viewers. In all the illustrations that follow the component patterns can be seen by viewing through one coloured filter at a time.

**Figure 4.**
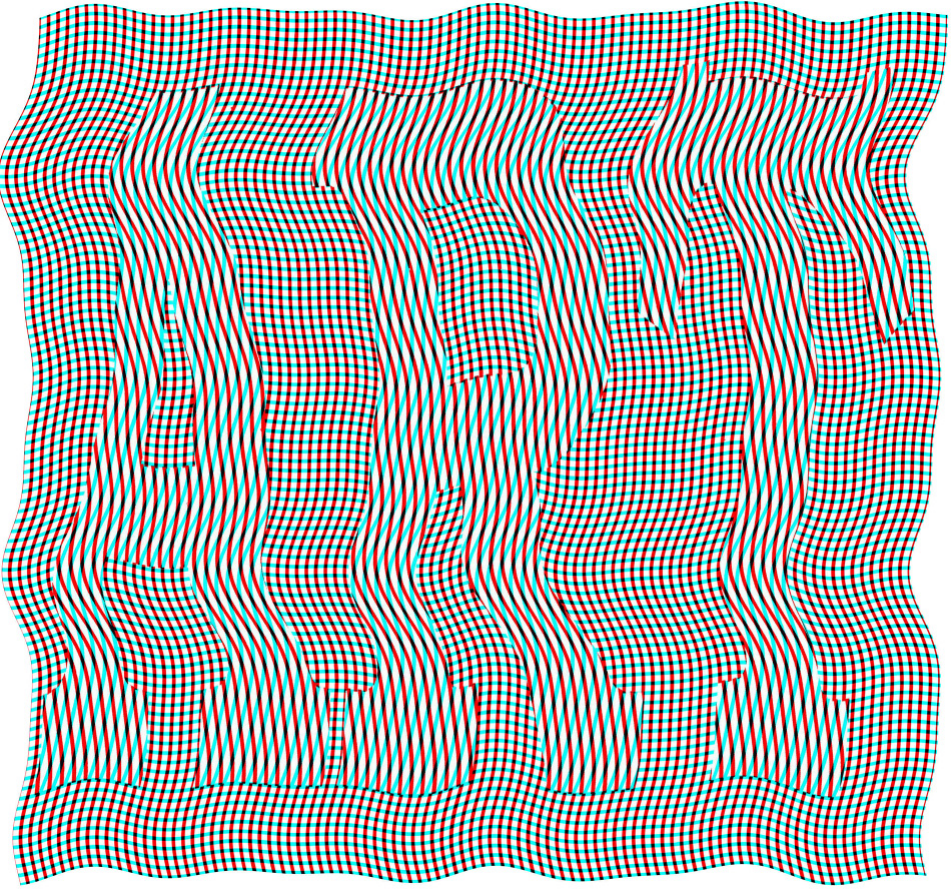
*Rivalry ART* by Nicholas Wade.

Figure 4 is a simple pattern of differently orientated, curved lines in each eye which engages in vigorous rivalry while retaining the visibility of the word ART. This is a consequence of the outlines of the letters (where the orientations change) being available to both eyes.

## Photographs in Rivalry

The first photographs that displayed rivalry were produced in error! The inventor of the stereoscope (Charles Wheatstone) asked the inventor of the negative/positive photographic process (William Henry Fox Talbot) to take stereoscopic photographs for him, but Talbot made the separations between the two views too large to be combined stereoscopically, resulting in rivalry. In a letter to Talbot, Wheatstone wrote:

I thank you for the photographs you have made for the stereoscope: they do not exactly answer the purpose as the angle you have taken (47½°) is too large and the differences in the two pictures are consequently too great, but they are sufficient to show that the effect when properly produced would be very good. 25° would be a much better angle. (http://www.foxtalbot.dmu.ac.uk/letters/transcriptName.php?bcode=Whea-C&pageNumber=6&pageTotal=24&referringPage=0)

**Figure 5.**
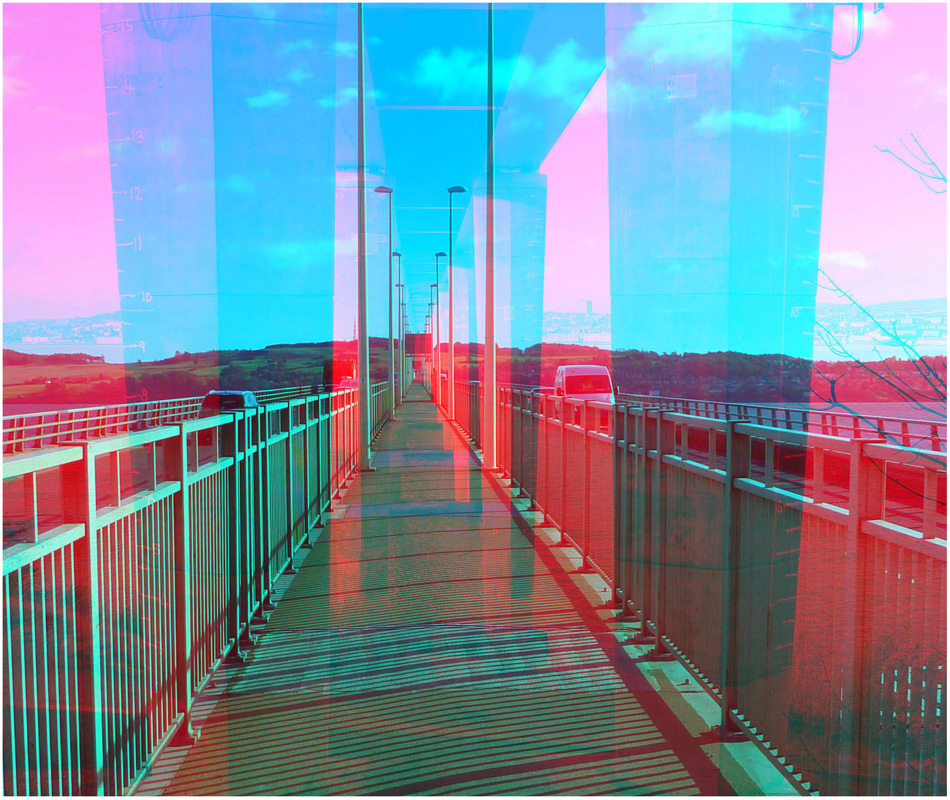
*Tay Road Bridge from above and below* by Nicholas Wade.

Unlike stereoscopic photographs, where the two components need to retain close correspondence in space and time, rivalling photographs are not so constrained. Moreover, there is no ‘correct’ arrangement of the filters and eyes: stereoscopic photographs do not reverse in depth with reversal of the filters but rivalry can be quite different with such reversals, depending on eye dominance. Rivalling photographs do require to be sufficiently different for competition rather than cooperation to take place. Another strategy is to combine two similar photographs with a few features in rivalry; the stability of the overall scene is disturbed by the localized rivalry within it. It is also possible to select the components so that they reflect different aspects of the same subject, as applies to the rivalling photographs in Figure 5. They are views of the same structure (a road bridge) from the central walkway and from beneath so that the supporting piers are seen.

**Figure 6.**
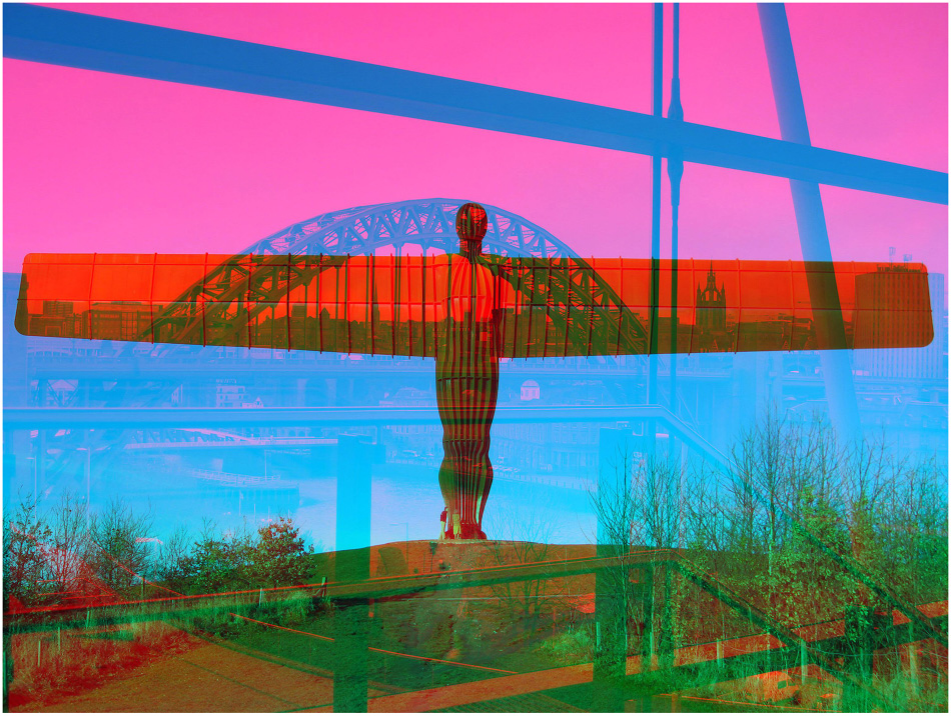
*Angel of Newcastle* by Nicholas Wade.

The structures of another bridge are shown in Figure 6. A photograph of the *Angel of the North* in Gateshead is presented to one eye and a view of the Tyne Bridge (taken from within the Sage Gateshead arts centre) to the other eye. The geometrical structures within the building contrast with the ironwork of the Tyne Bridge and the skyline of Newcastle is aligned with the outstretched wings of the angel.

**Figure 7.**
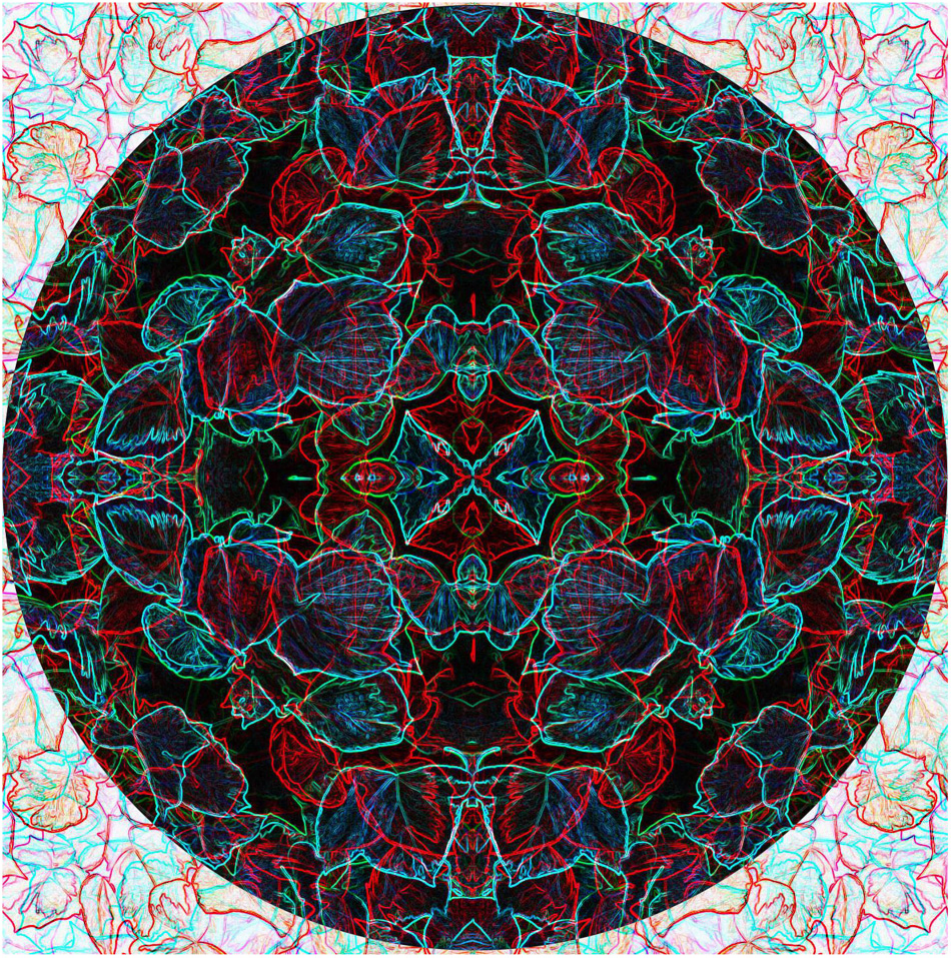
*Variegated rivylry* by Nicholas Wade.

The rivalling photographs can be manipulated prior to their combination as anaglyphs, as in Figure 7. A photograph of variegated ivy leaves was multiplied to create a symmetrical pattern which was then rendered into a line image. The pattern was combined with a copy rotated by 90° to form a rivalling anaglyph. The final image is a combination of the central circular negative of the combination together with a positive surround.

## Graphics in Rivalry

**Figure 8.**
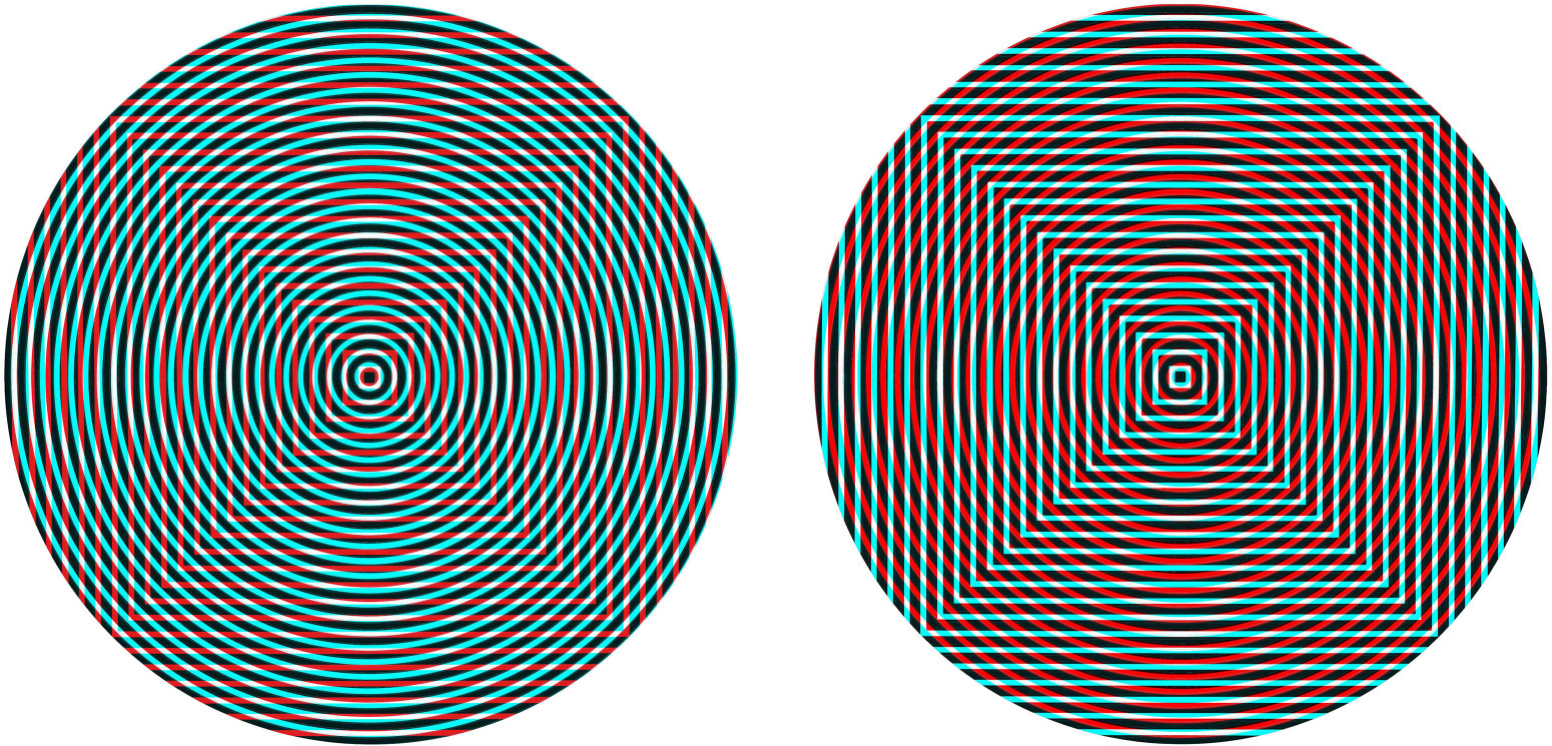
*Circling the squares* by Nicholas Wade.

Experimental research on binocular rivalry has tended to use gratings as stimuli, like those in Figure 1. Either achromatic or complementary-coloured parallel lines are presented to one eye with the same pattern at right angles in the other. A similar scheme is shown in Figure 8 which involves concentric circles and concentric squares. These are, however, more complex rivalling stimuli than gratings because the competing contours intersect at a range of angles that are not consistent throughout.

**Figure 9.**
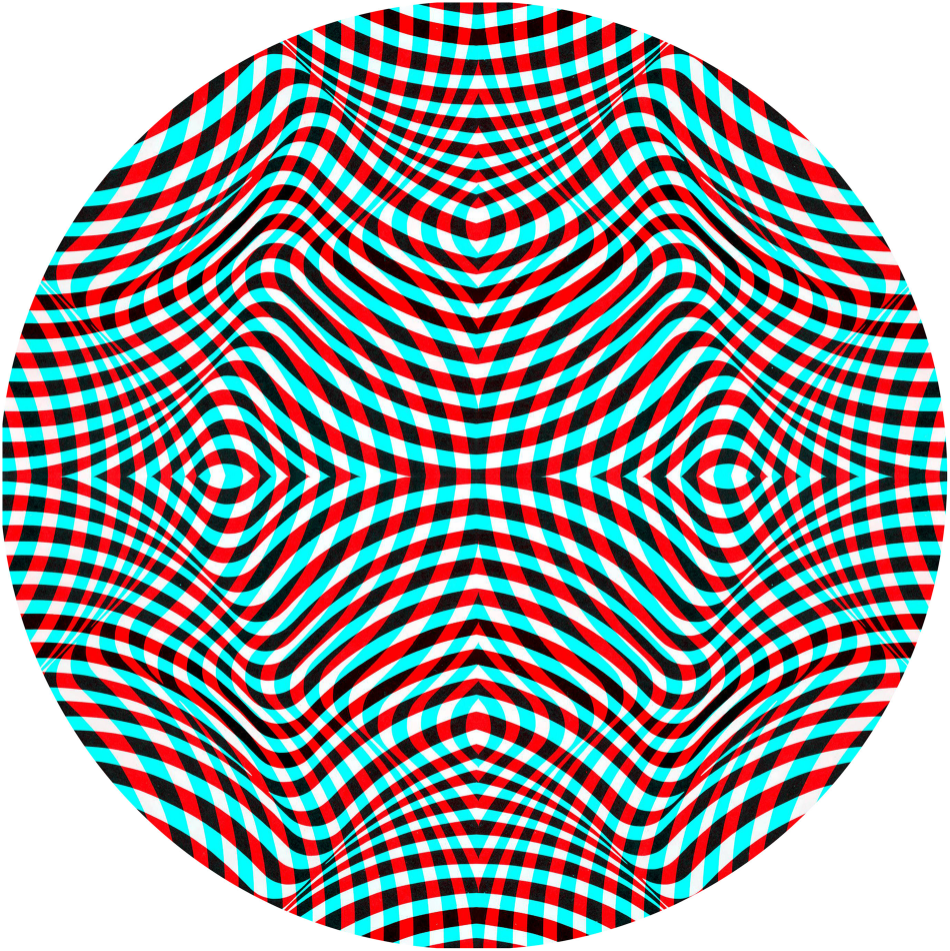
*Curvilinear rivalry* by Nicholas Wade.

A hallmark of art is to complicate rather than simplify the designs presented to observers and this applies to rivalling graphics, too. A more complex pattern is shown in Figure 9 where two symmetrical curved designs, resembling two eyes, are at right angles to one another. The monocular contours appear to be in depth, looking like humps and hollows, but this is not seen so readily when viewed binocularly due to the dynamic and piecemeal changes that are perceived.

**Figure 10.**
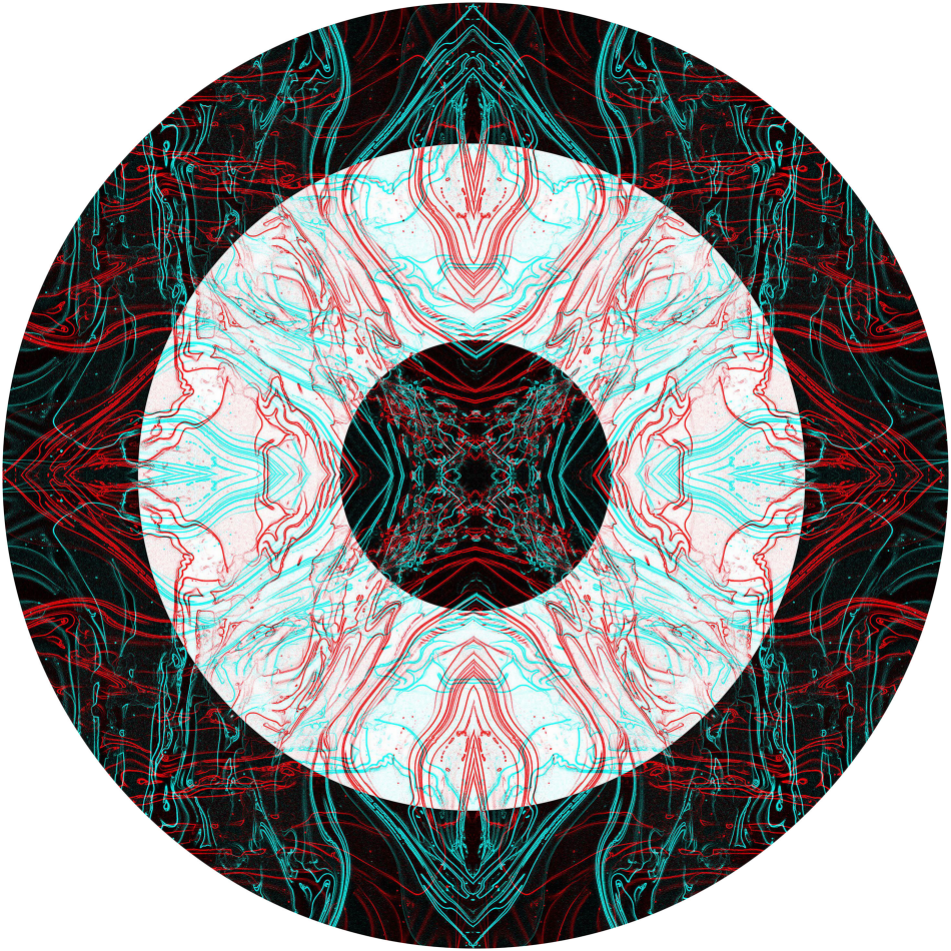
*Following the flow* by Nicholas Wade.

The graphical elements engaging in rivalry can be derived from a variety of sources. The components in Figure 10 have a long graphical history prior to combination as an anaglyph. The first stage consisted of an abstract painting made by a process similar to marbling; oil-based paints were dripped carefully onto water lying on a horizontal board which was then tilted around leaving a flowing pattern of intermingled coloured paint. When the water had evaporated and the paint dried, part of it was photographed, digitized and manipulated with computer graphics. The final anaglyphic image is a combination of rivalling negative and positive line images enclosed within one another. The same contours course continuously through the whole pattern changing from red to cyan when the meet the circular boundaries.

**Figure 11.**
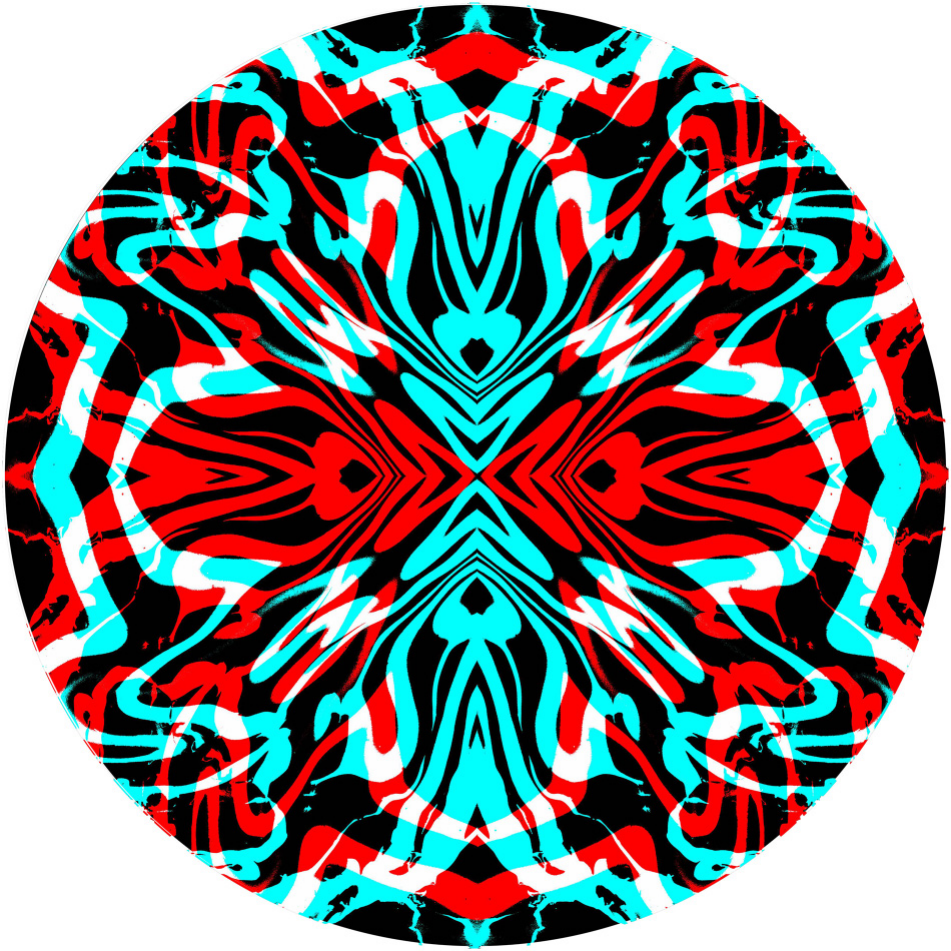
*Centripetal patterns* by Nicholas Wade.

In the graphical examples above, rivalry occurs throughout the patterns. In areas where contours are present in one region and not in those of the corresponding eye then the contours tend to predominate for most of the viewing time. This is not uniformly the case in Figure 11, where some regions have competing contours and others do not, resulting in more complex interactions between eye and pattern rivalry.

**Figure 12.**
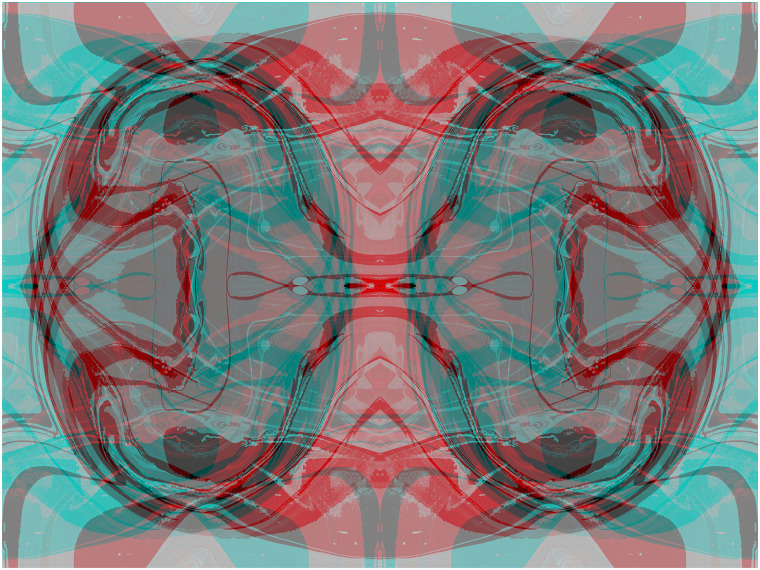
*Ogle* by Nicholas Wade.

Rivalry is rarely a subtle phenomenon and the paired patterns that are applied to express it are usually of high contrast. However, it is possible to use lower contrast designs to produce binocular competition but it might take a little longer for the rivalry between the patterns or parts of them to become visible. The components of the following pattern are themselves somewhat intricate and like those described for Figure 10. Paints suspended on the surface of a pool of water were flowed over a flat board, then allowing the different colours to intermingle and dry. The painting or details of it were photographed, digitized and manipulated in the computer. The digital images were usually multiplied and combined in order to produce the symmetries that are evident in them. Only at that stage was the anaglyphic image assembled.

## Combinations of Graphics and Photographs

**Figure 13.**
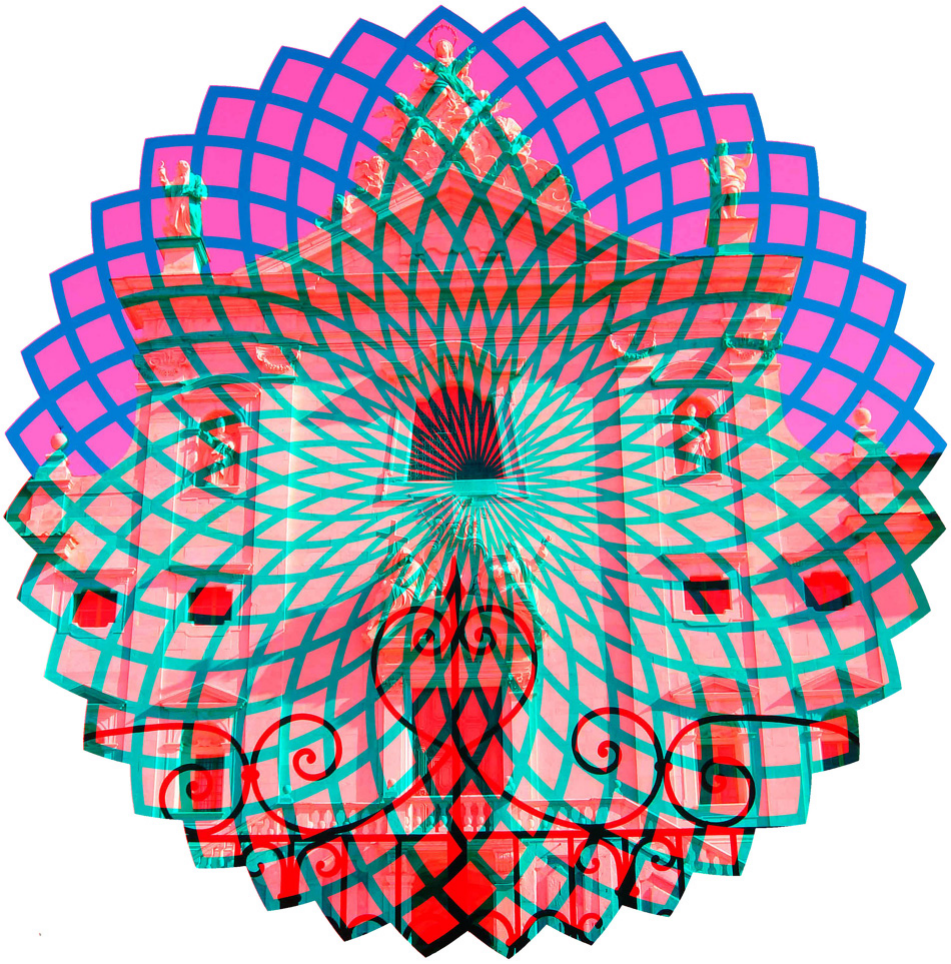
*Spiritual radiations* by Nicholas Wade.

**Figure 14.**
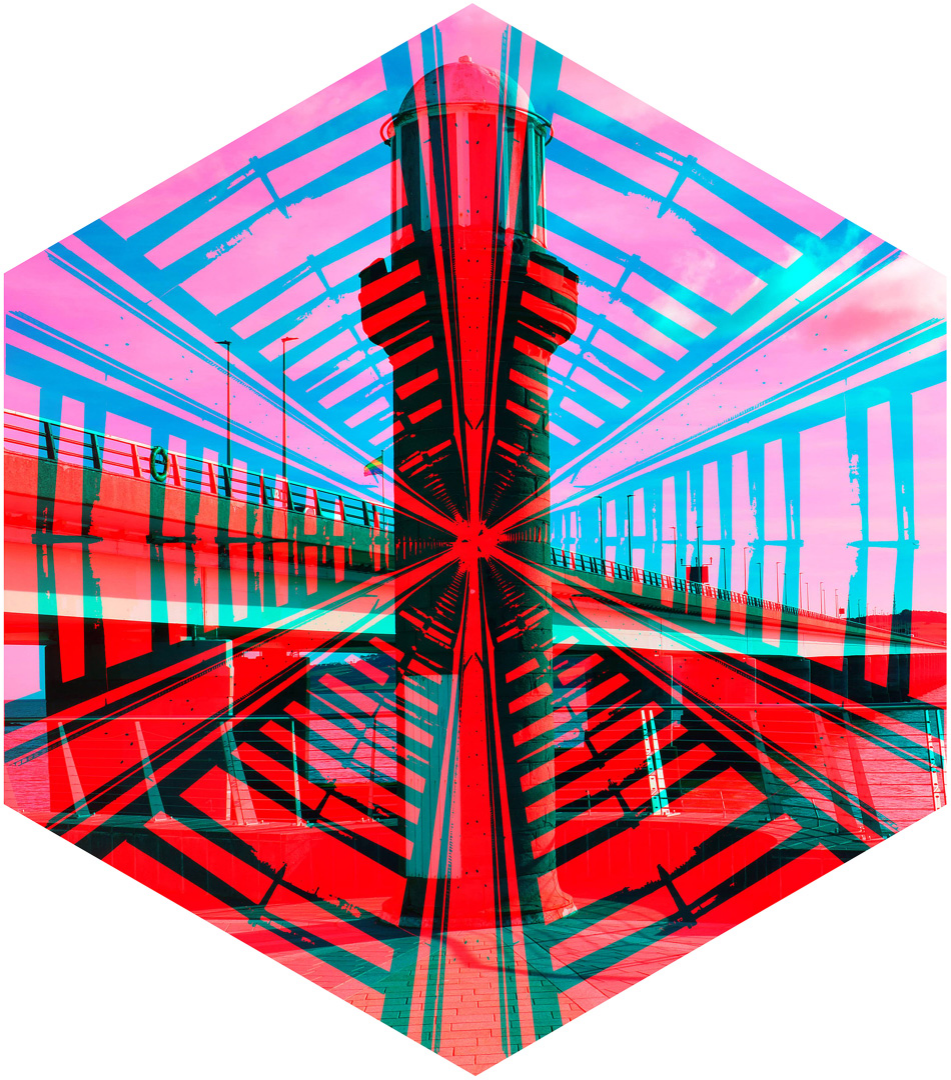
*Tay Road Bridge and Telford Light* by Nicholas Wade.

Rivalling contours can be derived from a variety of sources and the following anaglyphs combine photographs with graphics that have some relationship to them. In the case of Figure 13, a photograph of the gates and chapter house at Calci in Tuscany is combined with a radiating design. A perceptual effect is evident when the design alone is viewed: illusory dots can be seen at the intersections of the radiating spirals (the design is the negative of Figure 1.6.11 in Wade, 1982). Figure 14 shows in one eye a photograph of the Tay Road Bridge looking south with the Telford Light in the foreground and in the other a kaleidoscopic design based on the bridge seen from afar. In both figures the outline is defined by the graphical design.

**Figure 15.**
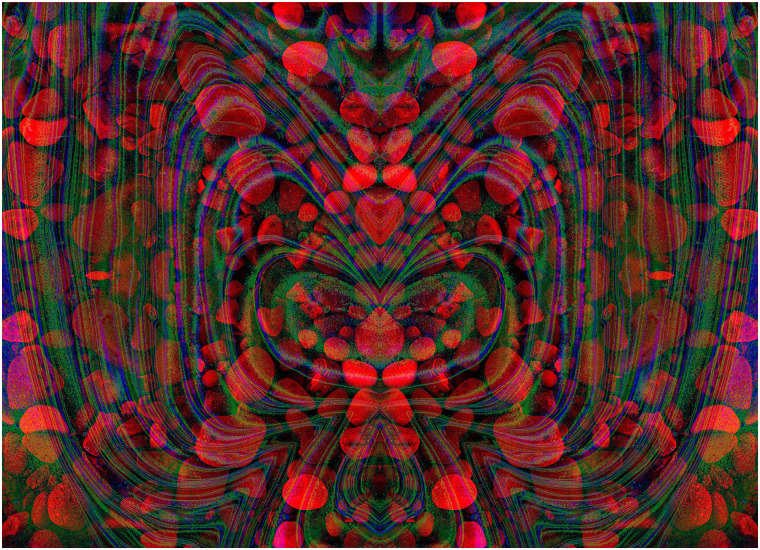
*Stones of destiny* by Nicholas Wade.

A more subtle design is in rivalry with a composite photograph of stones in Figure 15. The two components share symmetry about a central vertical axis. The photograph is composed of stones on a beach and the design is derived from an abstract painting using techniques similar to those described for Figures 10 and 12.

## Binocular Lustre

**Figure 16.**
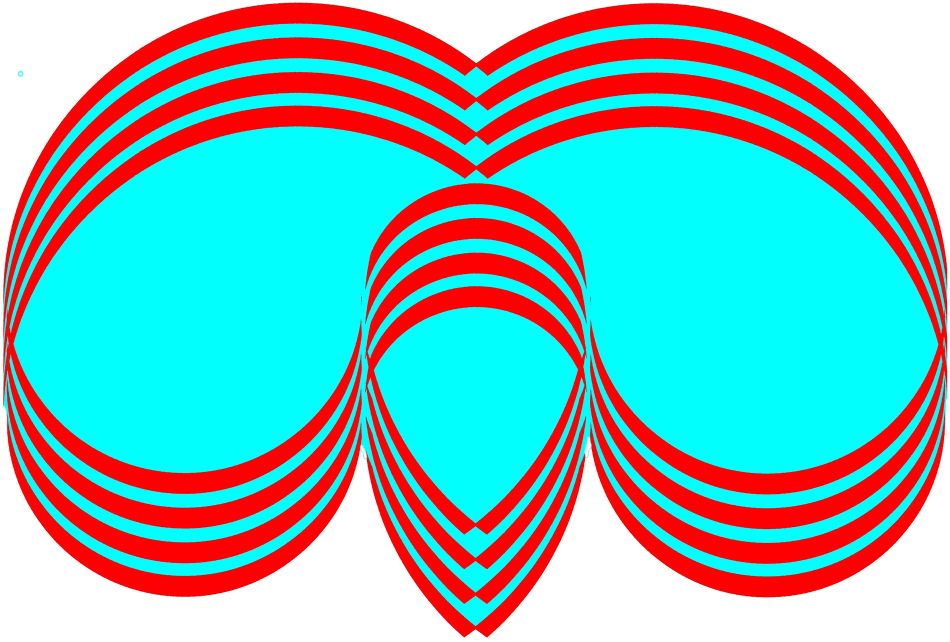
*Golden section arcs* by Nicholas Wade.

When a positive image is presented to one eye and its negative to the other the impression of a metallic sheen is called binocular lustre (see Wade, 2021a). The metallic sheen is more evident in larger areas devoid of contours as can be seen in Figure 16.

**Figure 17.**
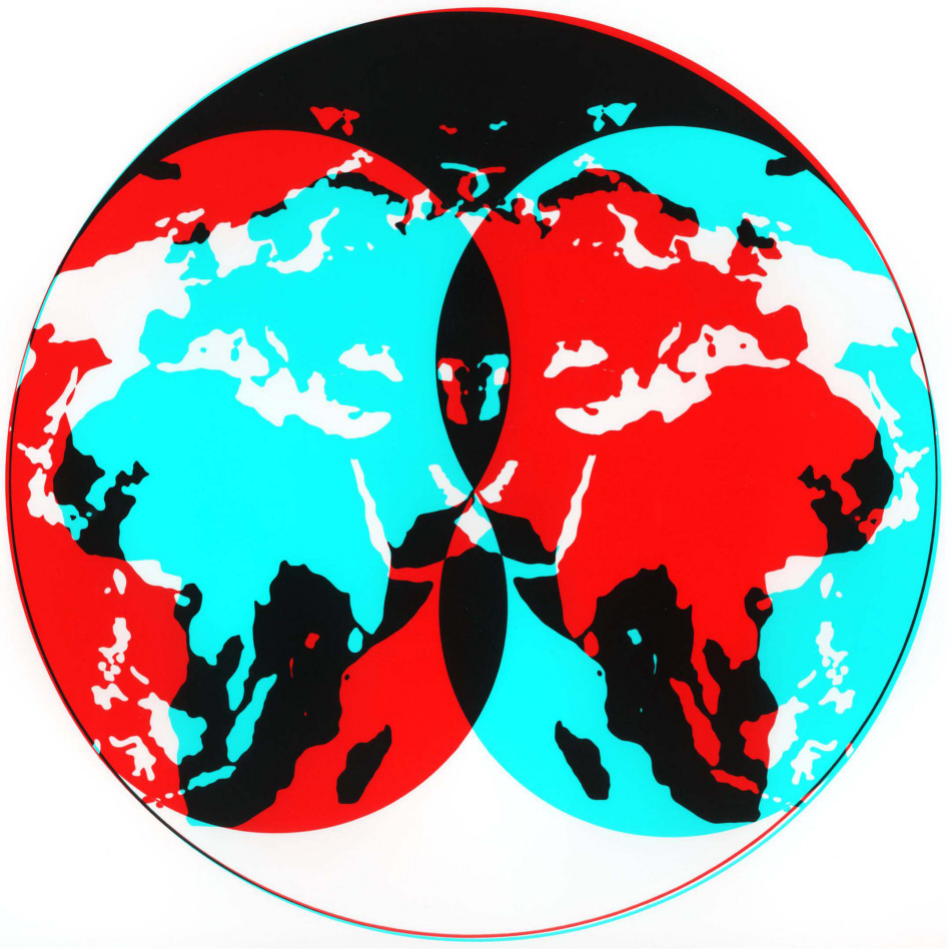
*The union of opposites* by Nicholas Wade.

Lustre can be expressed in photographs, graphics and their combination. Figure 17 contains global maps that are reversed with respect to one another; lustre can be seen in the overlapping regions and the binocular combination reveals the yin-yang designs from which each component is composed.

## Portraits in Rivalry

Portraits that rival with one another can be of the same person either in contrasting postures, at different ages or carried by appropriate graphical or textual motifs. The two components need not be of the same person so that a wide variety of possibilities can be entertained. Some rivalling anaglyphic portraits are illustrated in Wade (2019, 2021a, 2021c, 2022) and they include pioneers of research on binocular vision like Wheatstone and Brewster in rivalry as well as Helmholtz at different ages and Panum combined with orthogonal gratings.

**Figure 18.**
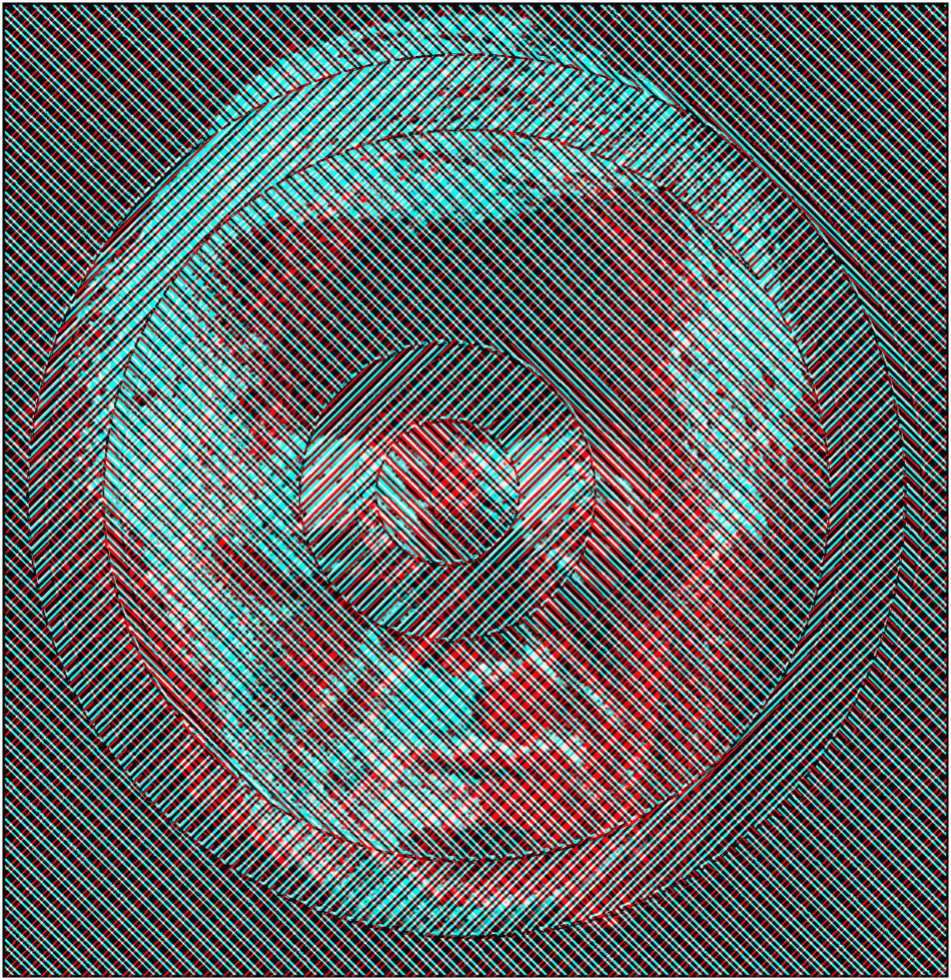
*Rivalry between Wheatstone and Brewster* by Nicholas Wade.

 Figure 18 shows the negative portraits of Wheatstone and Brewster in rivalry contained within patterns that are themselves in rivalry. Each monocular grating pattern is quite complex, involving concentric annuli defined by orthogonal contours Not only are the portraits and gratings in rivalry but there is also an additional graphical twist: the left and right central circle and annulus are displaced relative to one another and slight stereoscopic depth can be seen in them. Thus, there is a combination of rivalry and stereopsis (see Andrews and Holmes, 2011; Riesen et al., 2019).

**Figure 19.**
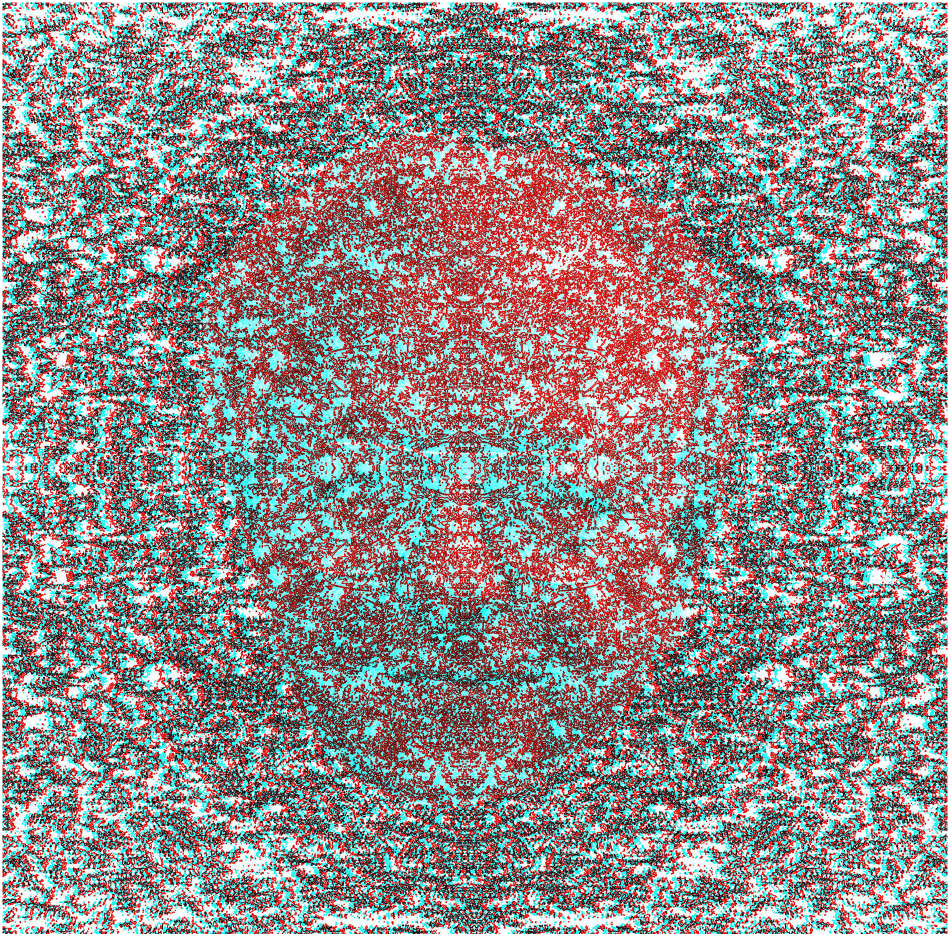
*Bela Julesz in rivalry* by Nicholas Wade.

Research on stereoscopic depth perception was transformed by the introduction of random dot stereograms by Julesz (1960, 1971) and its influences rippled through pictorial art, too (see Wade, 2021b). It was possible to examine stereopsis without monocular cues for the appearance of depth. The technique was not pursued in the context of binocular rivalry but it can be. Figure 19 displays both stereoscopic depth and rivalry using a naturalistic ‘carrier pattern’ (derived from a photograph of a gorse bush) rather than computer generated random dots. Julesz's portrait can be seen in one eye but with two eyes the region in depth is visible as well as evanescent appearances of the portrait. The circular region surrounding the portrait reverses in depth with reversal of the filters.

**Figure 20.**
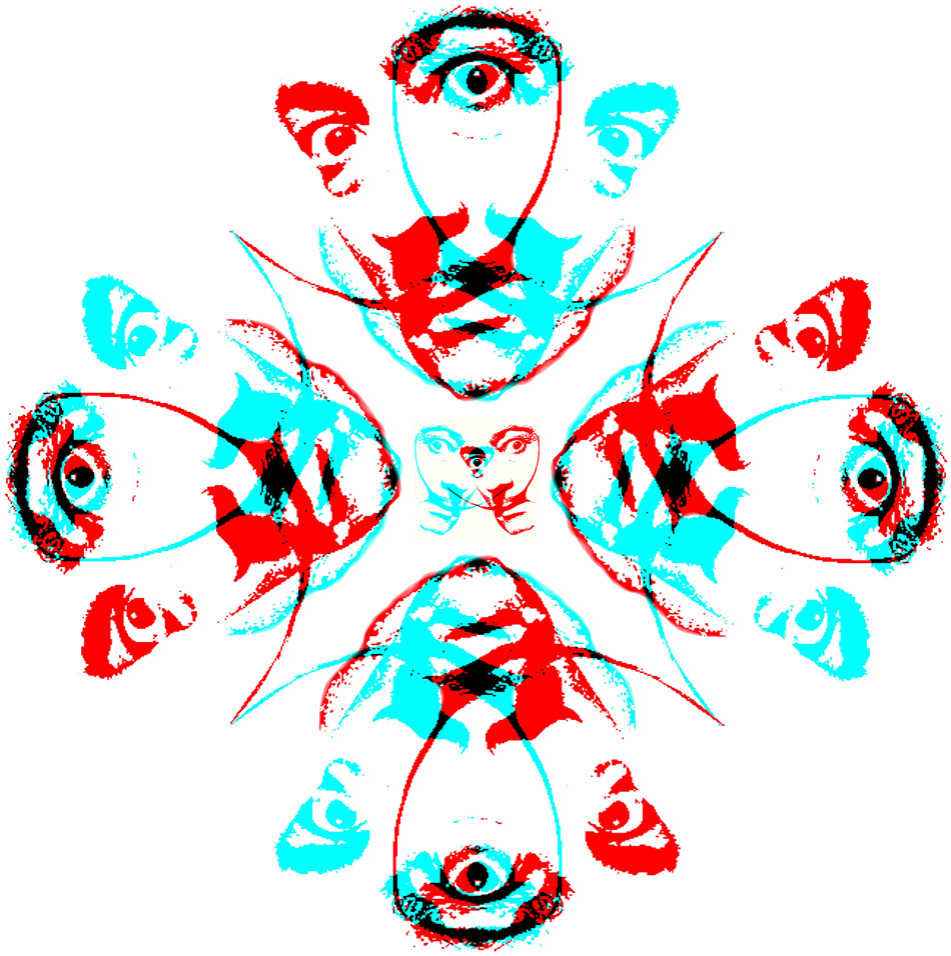
*Salvador Dali’s third eye* by Nicholas Wade.

Some artists have experimented with stereoscopic depth and Salvador Dali is the most prominent amongst them (Ades, 2000). Both Dali and Marcel Duchamp made works in which the left and right eye images were radically different and engaged in binocular rivalry (Ades, 2008; King, 2018). A manipulated portrait of a moustachioed Dali in partial rivalry is shown in Figure 20. The four peripheral double portraits are connected with the tips of his trademark moustache and each overlapping face shares a central eye which is in rivalry. The eye common to the four outer paired portraits is different to that shared in the smaller central pair.

**Figure 21.**
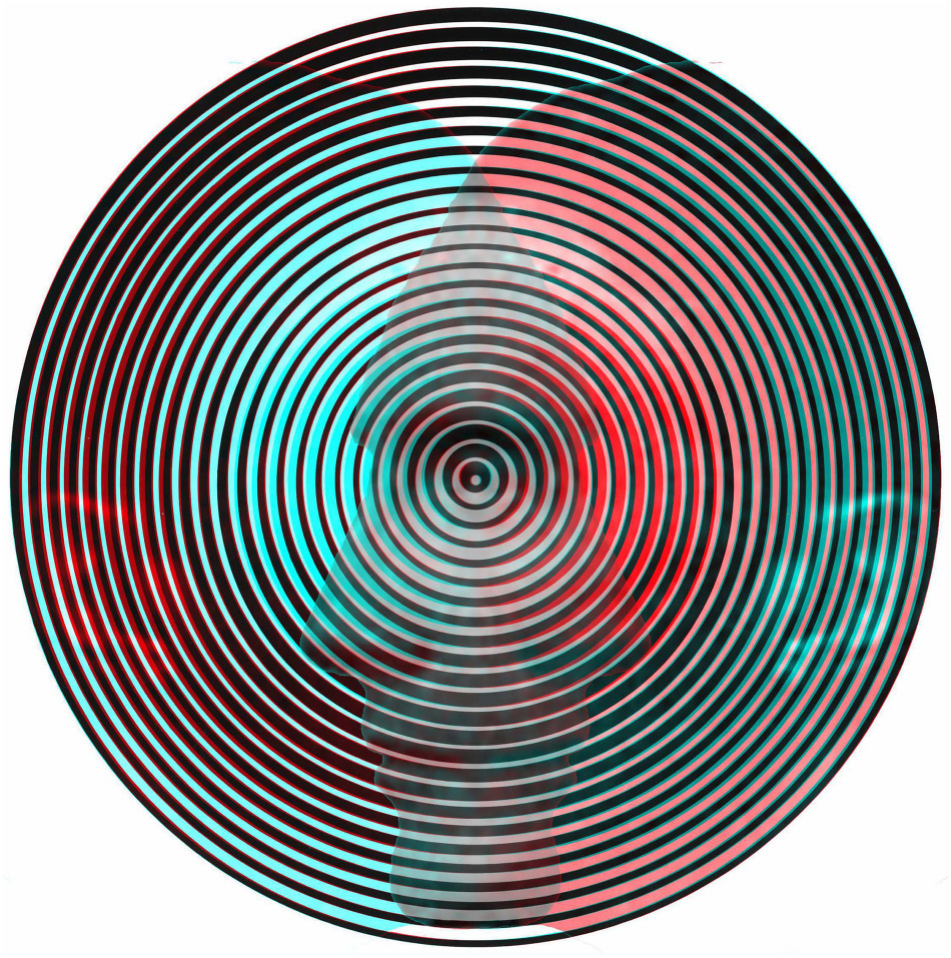
*Double Duchamp* by Nicholas Wade.

Duchamp produced a range of binocular works some of which involved additions by hand to existing stereoscopic photographs and others were anaglyphs (King, 2018). Duchamp's double portrait in Figure 21 displays binocular rivalry and stereoscopic depth. The two profiles share a central eye which appears to approach or recede dependent on the arrangement of filters before the eyes. The circular design in which the profiles are embedded is rather like some of the rotoreliefs Duchamp produced after his stereoscopic experiments: eccentric circular patterns can appear in depth when rotated slowly (see Mannoni et al., 2004; Wade, 1982, 2016a).

**Figure 22.**
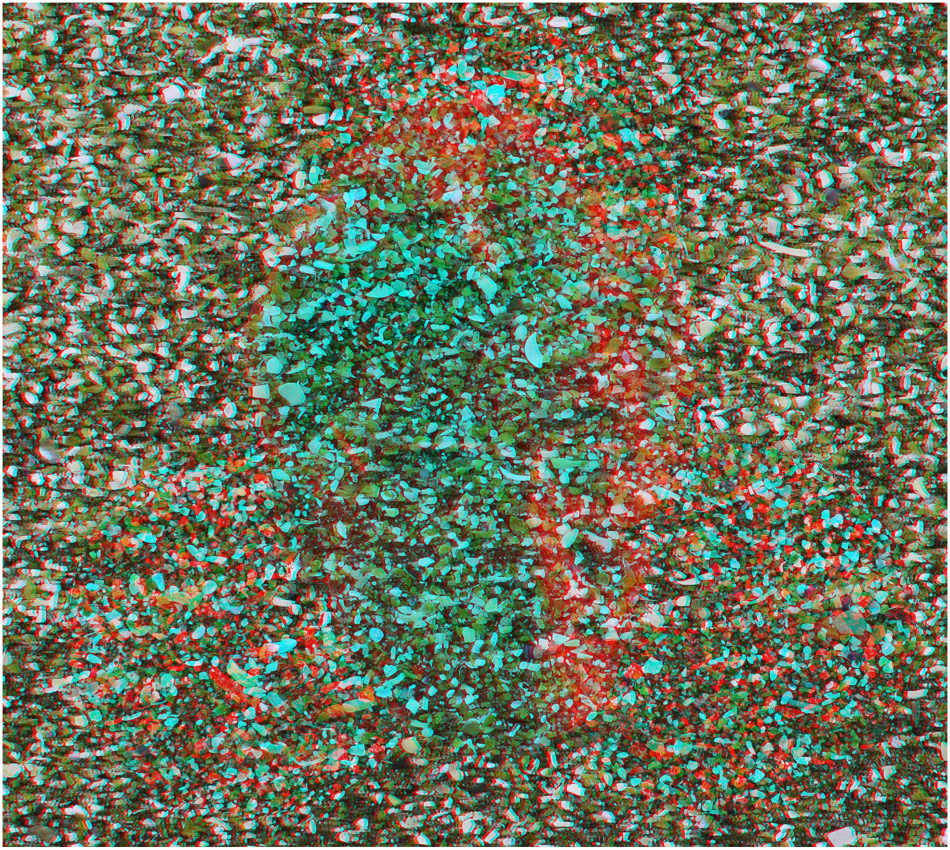
*The triangle of Kanizsa* by Nicholas Wade.

The technique of combining stereopsis and rivalry can be applied to illusory figures and their creators. Figure 22 involves a stereoscopic Kanizsa triangle and a portrait of Gaetano Kanizsa himself. The carrier pattern for the stereoscopic sectored circles is derived from a photograph of shells on a beach. Kanizsa was a psychologist in the Gestalt tradition and he renewed interest in subjective contours in the 1970s with his illustrations, particularly with his triangle figure (see Kanizsa, 1979). He was also an accomplished artist and his abstract, largely black and white, paintings were widely exhibited in Italy. His portrait, with his rueful smile and his shock of hair, is at the margins of visibility and competes with the missing sectors of the three stereoscopic circles.

**Figure 23.**
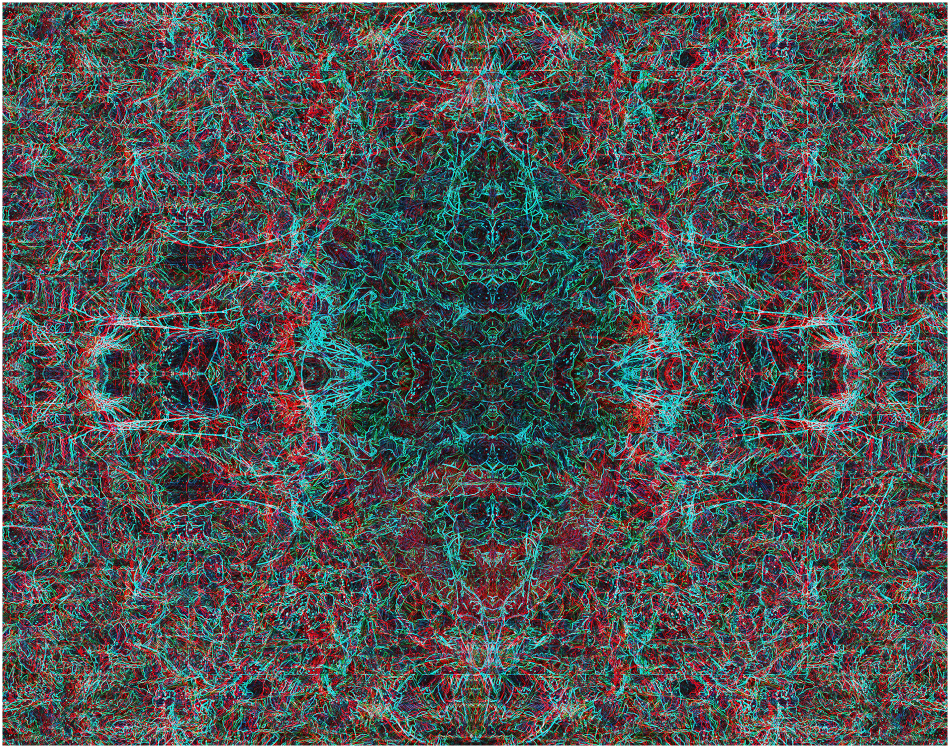
*Self-portrait in rivalry and depth* by Nicholas Wade.

The art of binocular rivalry can be expressed in many ways but perhaps the most attractive is the interplay between binocular cooperation and competition, as is evident in Figure 23.

## Conclusions

Wheatstone (1852) predicted that the marriage of the camera and stereoscope would influence the course of art and so it has proved. However, this influence tends to have been restricted to stereoscopic depth perception rather than binocular rivalry. This could reflect the peripheral part that rivalry plays in our perception. Binocular rivalry is a natural consequence of our binocular interactions with the world; rivalry is a resolution of conditions that apply to most of what we see when using two eyes. It occurs when the differences between the images in the two eyes are too large to be combined, and stereoscopic depth cannot be extracted from disparity. When we fixate with both eyes on an object most of what is projected to the peripheral retina is too disparate to yield depth; since the peripheral stimuli arise from different depths to those fixated their retinal images also tend to be out of focus. We are not generally aware of this binocular rivalry as both visual resolution and attention are associated with the fixated object rather than peripheral ones. Binocular rivalry is rarely examined under these conditions of natural stimulation. It is typically studied with different patterns presented to corresponding foveal regions of the two eyes – as if we are fixating on two different objects at the same time. Under these conditions our vision is unstable and it is this state that has been the subject of much scientific enquiry but it has resulted in little art.

It could be argued that our preoccupation with pictures in art is as odd as the concern with binocular rivalry in science – both present stimuli that are rarely encountered in the natural environment. However, it is for this very reason that representational art has proved so alluring throughout recorded history – marks on a flat surface allude to objects that are not present. The fate of binocular rivalry has been different – there are no objects to which the fluctuating visibility can refer. The case could be otherwise for abstract art which is associated with more basic visual processes than object recognition but even here this dynamic aspect of our vision has not been widely recognised:

Binocular rivalry has not previously been incorporated within the armoury of Op Artists, but it seems particularly suited for inclusion – constant variations in perception are provided by the operation of the visual system itself without further intervention of the artist or scientist. (Wade, 1982, p. 3)

Developments in stereoscopic techniques and a realization of the intriguing effects that are open to manipulation might encourage more artists to engage with binocular rivalry.
